# Harnessing ResNet50 and SENet for enhanced ankle fracture identification

**DOI:** 10.1186/s12891-024-07355-8

**Published:** 2024-04-01

**Authors:** Hua Wang, Jichong Ying, Jianlei Liu, Tianming Yu, Dichao Huang

**Affiliations:** 1 Department of Medical Imaging, Ningbo No. 6 Hospital, Ningbo, China; 2 Department of Orthopedics, Ningbo No. 6 Hospital, Ningbo, China

**Keywords:** Ankle fractures, Deep learning, ResNet50, SENet, Radiographic images, Grad-CAM

## Abstract

**Background:**

Ankle fractures are prevalent injuries that necessitate precise diagnostic tools. Traditional diagnostic methods have limitations that can be addressed using machine learning techniques, with the potential to improve accuracy and expedite diagnoses.

**Methods:**

We trained various deep learning architectures, notably the Adapted ResNet50 with SENet capabilities, to identify ankle fractures using a curated dataset of radiographic images. Model performance was evaluated using common metrics like accuracy, precision, and recall. Additionally, Grad-CAM visualizations were employed to interpret model decisions.

**Results:**

The Adapted ResNet50 with SENet capabilities consistently outperformed other models, achieving an accuracy of 93%, AUC of 95%, and recall of 92%. Grad-CAM visualizations provided insights into areas of the radiographs that the model deemed significant in its decisions.

**Conclusions:**

The Adapted ResNet50 model enhanced with SENet capabilities demonstrated superior performance in detecting ankle fractures, offering a promising tool to complement traditional diagnostic methods. However, continuous refinement and expert validation are essential to ensure optimal application in clinical settings.

## Introduction

### Overview of ankle fractures

Ankle fractures are traumatic injuries that predominantly afflict the lower extremities. Recent epidemiological data reveals that ankle fractures account for roughly 15% of all adult fractures each year, showcasing their significant incidence rate [1]. These injuries are commonly attributed to a twisting or rolling motion of the ankle. The increasing global prevalence of such injuries is speculated to arise from a combination of heightened physical activity and an aging demographic [2].

The categorization of ankle fractures often leans on the severity of the injury, which inherently dictates the corresponding therapeutic strategy. Mild fractures, characterized by minor cracks, may sometimes elude detection on standard X-rays. For these, the RICE protocol (Rest, Ice, Compression, and Elevation) combined with immobilization using a brace or cast is frequently the chosen course of treatment [3]. Moderate fractures, which stand as an intermediate category, can demand longer immobilization durations and occasionally minor surgical interventions. This is especially true when the injury is accompanied by a minor dislocation or indicates more profound structural disruption [4]. In contrast, severe fractures, which are marked by substantial bone displacement, invariably necessitate surgical procedures to realign the bones and stabilize the joint. Recovery from such fractures usually entails rigorous rehabilitation to recover complete function and avoid potential complications [5].

Distinguishing ankle fractures from other potential injuries, such as sprains, is a nuanced and often challenging endeavor. Conventional X-rays are the primary diagnostic tool for these fractures. However, their sensitivity, especially concerning potential hairline fractures or occult injuries, is not foolproof. In scenarios where the X-rays yield inconclusive results, other imaging modalities, like computed tomography (CT) scans or magnetic resonance imaging (MRI), are employed to facilitate a more definitive diagnosis [6].

The adverse outcomes stemming from inadequately diagnosed or untreated ankle fractures cannot be understated. Beyond the immediate pain and functional impairment, these fractures, if misdiagnosed, can be a precursor to conditions such as post-traumatic arthritis [7]. This, in turn, can severely hamper mobility and might necessitate more aggressive interventions in the long run. The cumulative impact of such oversight not only exacerbates the physical ailment but also intensifies the psychological and economic burdens on the affected individuals.

### Overview of using machine learning and CNNs for ankle fractures diagnosis

Despite advances in imaging technology and treatment options, the diagnosis of ankle fractures remain challenging. One potential solution to this problem is the use of machine learning algorithms to assist in the diagnosis and classification of ankle fractures. Machine Learning is a field of artificial intelligence (AI) that uses algorithms and techniques to enable machines to learn from data rather than being explicitly programmed [8]. It involves algorithms that can detect patterns in data, classify data, and make predictions [9]. The methods used to implement Machine Learning are known as Machine Learning methods, which include supervised learning, unsupervised learning, semi-supervised learning, reinforcement learning, and deep learning [10]. Machine learning (ML) methods have become increasingly popular in various application areas [11], with Random Forest (RF), Support Vector Machine (SVM), eXtreme Gradient Boosting (XGBoost) and Convolutional Neural Networks (CNNs) being some of the most commonly used [12].

The use of machine learning in orthopaedics is becoming more and more widespread, as demonstrated by the research of Lalehzarian et al. (2021) [13]. In particular, the application of machine learning to the diagnosis and treatment of ankle fractures is gaining traction [14, 15]. By utilizing patient data and imaging scans, machine learning algorithms can accurately predict the probability of an ankle fracture and its severity. Moreover, machine learning can be used to monitor the healing process and create personalized treatment plans. This can result in shorter recovery times and better outcomes for patients, as it allows doctors to customize their approach to the specific needs of the patient. To date, convolutional neural networks (CNNs) have been widely applied to medical image recognition, with Yang et al. (2022) proposing a two-stage CNN to detect scaphoid fractures [16]. Jaderberg et al. (2014) developed methods to reduce the computational cost of CNNs, allowing for greater deployment of these powerful models [17]. Kitamura et al. (2019) attempted to train CNN models de novo using a small dataset of 596 normal and abnormal ankle cases [18]. Derkatch et al. (2019) used twelve 742 routine clinical Vertebral Fracture Assessment (VFA) images for CNN training and testing [19]. Sinha et al. (2020) used radiographs obtained from 1050 patients with ankle fracture and the same number of healthy individuals to train deep convolutional neural networks (DCNNs) for dimple detection and segmentation in Titanium alloys [20]. However, the challenge of using CNNs for ankle fracture identification lies in the complexity of the task, as well as the need for a large and diverse dataset of images that represent a wide range of ankle fracture types. Additionally, the model must be able to generalize to unseen examples and provide reliable and accurate predictions in order to be useful in clinical practice.

In light of recent advancements in deep learning for ankle fracture detection, studies [21] and [22] have demonstrated the efficacy of employing deep convolutional neural networks (DCNNs) with radiographic images. Study [21] reported a high sensitivity of 98.7% and specificity of 98.6% using Inception V3 and ResNet-50 on radiographs, emphasizing the potential of DCNNs to accurately identify fractures from multiple views. Similarly, study [22] achieved notable accuracy and AUC values (up to 90%/0.95) in detecting ankle fractures using X-rays, further validating the capability of deep learning models in enhancing diagnostic precision with accurately labeled datasets.

### Research purposes

In light of the prior studies which have predominantly utilized CNNs in medical image recognition, our intention is to embark on a more nuanced approach. By employing the ResNet50 model, enriched with the capabilities of the Squeeze-and-Excitation Network (SENet), we aim to establish a model that is tailored to the specific challenges posed by ankle fracture identification. Moreover, recognizing that a model’s true efficacy is gauged by its capacity to generalize across unfamiliar samples and consistently produce dependable results, our research will juxtapose our adapted ResNet50 model against the original ResNet50 model and the EfficientNetB5 model. This comparative analysis seeks to discern the most adept classification model that can seamlessly be integrated into clinical practices.

Furthermore, our research will not solely be confined to the realm of classification. To amplify the diagnostic precision, we will integrate Grad-CAM technology, introducing a heatmap functionality. This innovative feature aims to pinpoint the exact regions of pathology, ensuring that medical professionals are equipped with a comprehensive understanding of the fracture’s characteristics.

## Methods

### Data collection

In this study, we adopted a meticulous approach to data acquisition, leveraging a collaborative partnership with a local hospital to access their archive of ankle CT images. These single-channel, grayscale DICOM images, standardized at a resolution of 512 × 512 pixels and spanning three distinct views, formed the crux of our dataset. To ensure data quality, each image underwent stringent vetting by a panel of orthopedic experts. Out of numerous images evaluated, 987 were deemed apt for our research, segmented into 255 images depicting fractures and 732 illustrating normal ankles. Having secured permissions from the hospital, we synchronized with the medical staff to cull images that encapsulated both quality and relevance to ankle fractures. Once curated, these images were uniformly converted to the DICOM format and stored securely. Each image was then evaluated for diagnostic accuracy, with our panel of physicians distinguishing them into fractured or normal categories.

In this study, we elected to utilize CT images as the primary source for ankle fracture identification, considering their superior detail and three-dimensional reconstruction capabilities. CT scans offer enhanced clarity in delineating complex anatomical structures and are invaluable in cases where radiographic images may yield inconclusive results. The choice was also influenced by the standardized nature of our CT image dataset, which aids in model training by providing consistent image quality and dimensions. While radiographs are a fundamental tool in orthopedic diagnosis, their variability and the subtlety of certain fractures present significant challenges for automated analysis. This approach aligns with our goal to enhance the precision of fracture identification using machine learning, acknowledging the pivotal role of both CT scans and radiographs in clinical assessments.

Central to our research ethics, informed consent was paramount. Participants were briefed comprehensively about the study’s scope, potential risks, and benefits, ensuring their explicit consent before incorporating their images into our dataset.

### The proposed system

**Fig. 1:**
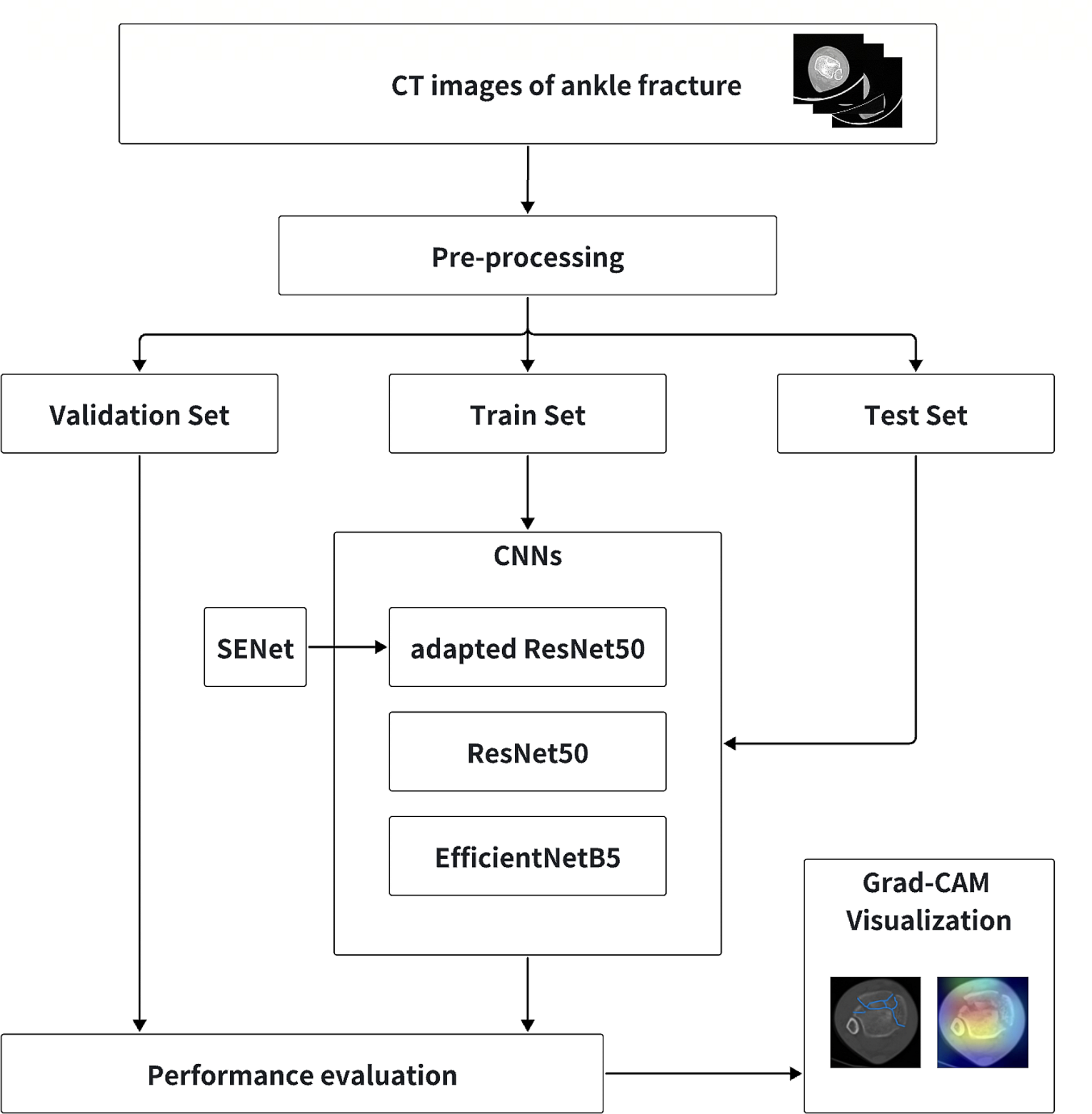
Flowchart of the machine learning-based diagnostic system for ankle fractures

In this study, we introduce a structured approach to enhance the diagnosis of ankle fractures using machine learning techniques integrated with medical imaging, as shown in Fig. 1. The initial phase involves obtaining CT images of ankle fractures, followed by a critical pre-processing step to standardize and refine the dataset. This pre-processed data is then partitioned into a training set (60%, 591 images), test set (20%, 198 images), and validation set (20%, 198 images) to ensure robust model training and evaluation.

A critical aspect of our dataset preparation was ensuring that images from the same patient were exclusively allocated to one cohort—training, testing, or validation—to prevent data leakage and uphold the integrity of our model’s evaluation. This allocation strategy was integral to our methodical approach, safeguarding the validity of our findings and the reliability of our deep learning model for ankle fracture identification.

The heart of our system lies in the application of Convolutional Neural Networks (CNNs), specifically utilizing the ResNet50 architecture. To address the challenges specific to ankle fracture identification, we augment ResNet50 with the Squeeze-and-Excitation Network (SENet), aiming for enhanced feature extraction. For a comprehensive analysis, the performance of the augmented ResNet50 is contrasted with the standard ResNet50 and EfficientNetB5 models, determining the most suitable model for clinical implementation.

Furthering diagnostic precision, we incorporate Grad-CAM technology into our system. This addition facilitates a heatmap visualization, pinpointing the exact regions of pathology, thus providing medical professionals with a more granular understanding of fracture characteristics. Overall, our proposed system offers a cohesive and academically rigorous approach to improve ankle fracture diagnosis through advanced machine learning models and diagnostic visualizations.

### Pre-processing

**Fig. 2:**
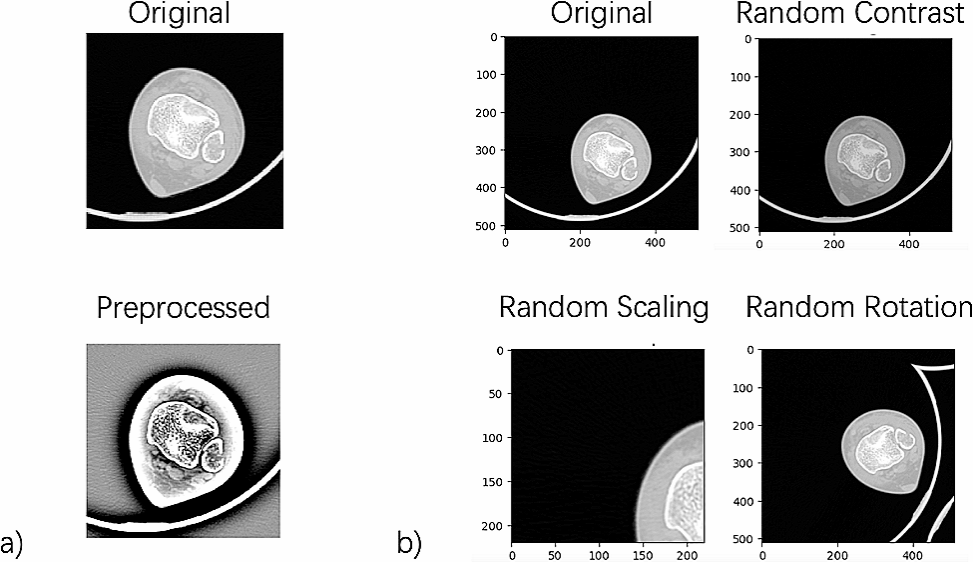
Data pre-processing: (2**a**) original vs. preprocessed CT image of a positive fracture case; (2**b**) example of data augmentation techniques applied

To optimize the efficiency of labeling and segmentation, images had to be preprocessed to meet the input size specification of CNNs with fully connected layers. Thus, the images were resized to a uniform size to ensure consistency in dimensions across the dataset. Utilizing Pydicom, each CT image was resized to a 224 × 224 pixel image and then converted to a vector matrix. Additionally, grayscale masks were added to the images to highlight the region of interest (ROI). This technique removed any irrelevant background information and focused the model’s attention solely on the bone. To simulate noisy and low-quality images that may be encountered in a clinical setting, Gaussian noise was added to the images. Data enhancement techniques such as data augmentation were also used to increase the size of the dataset and to reduce overfitting. Normalization was also applied to the images, which involved scaling the pixel values to a range of 0 to 1. This enabled the model to learn more efficiently by reducing the effect of varying intensity levels in the images. Finally, data augmentation involved generating new images from the existing ones by applying random transformations such as random contrast, random scaling, random rotation. After data augmentation, our dataset expanded to include 2,364 images for the training set, 792 images for the test set, and 792 images for the validation set. Figure 2 presents the original image and the preprocessed image of a positive fractures case, and the data augmentation action in this study.

### The integration of Squeeze-and-Excitation Networks with ResNet50

Squeeze-and-Excitation Networks (SENet) emerged as a pivotal advancement in the realm of deep learning, presenting a novel approach to model recalibration techniques [23]. Designed to adaptively recalibrate channel-wise feature responses, SENet aimed to enhance the representational power of a network without adding significant computational burden [24]. This approach’s fundamental premise was to focus on channel interdependencies to capture richer contextual information in image processing tasks, something classical CNN architectures often overlooked.

ResNet50, on the other hand, was part of the ResNet family - a series of deep residual networks recognized for their profound depth, which sometimes surpassed hundreds of layers [25]. This depth, while instrumental in capturing intricate patterns in images, was plagued by the vanishing gradient problem. However, the introduction of skip or shortcut connections in ResNet provided an avenue for the direct transmission of the gradient, alleviating the aforementioned issues.

**Fig. 3:**
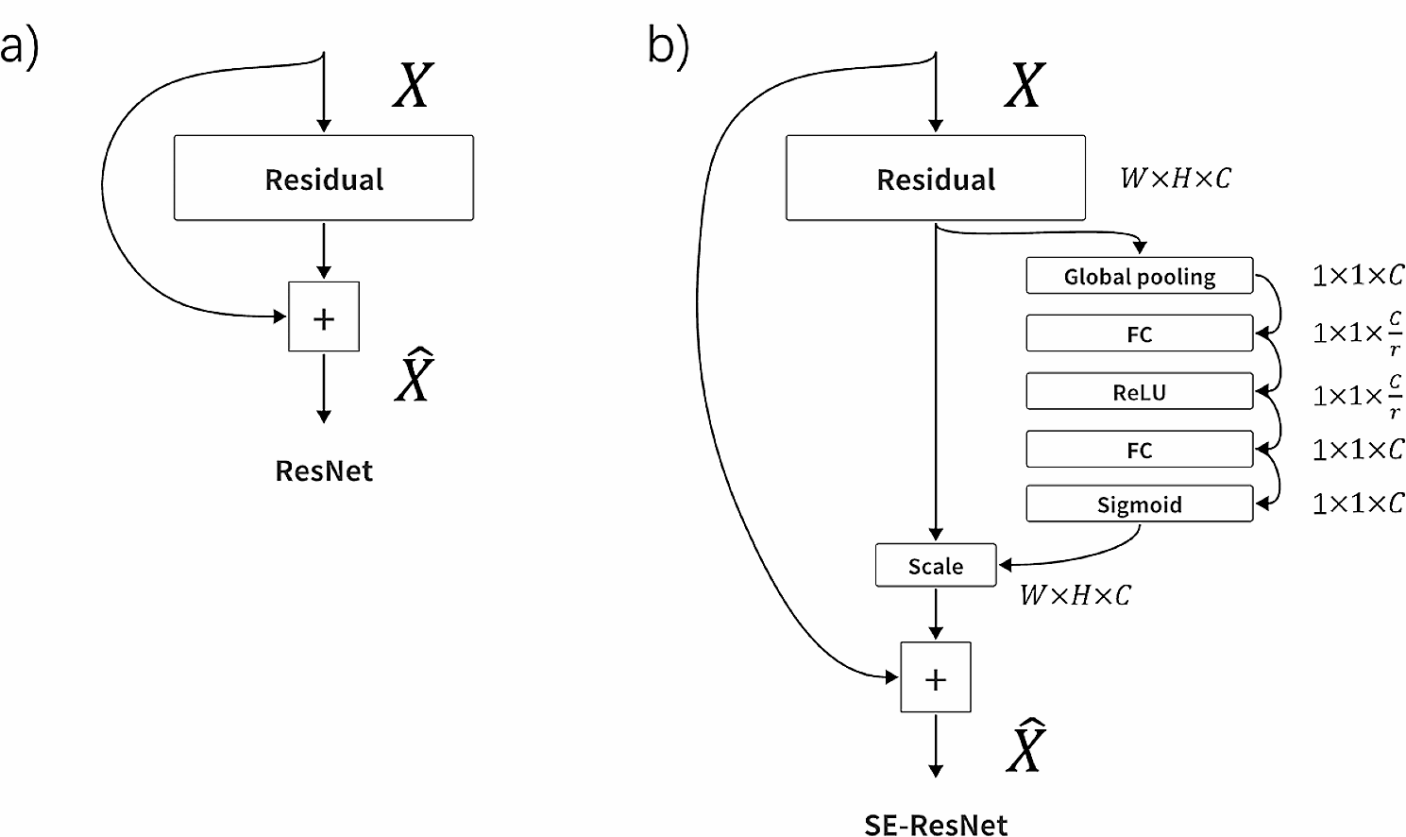
Architectural comparison of neural modules —(3**a**) traditional ResNet module and (3**b**) adapted ResNet module

Recognizing the distinct advantages of both SENet and ResNet50, our endeavor centered on the amalgamation of these two state-of-the-art technologies. During the establishment of the combined SENet + ResNet50 model, the primary challenge was integrating the recalibration mechanism of SENet into the deep residual framework of ResNet50. Figure 3 shows the integration approach of our model. The foundational ResNet module, shown on the left side of Fig. 3, uses an input “X” that undergoes transformation within a residual block. The benefit of ResNet lies in its skip connections: the input feature map can bypass certain layers, only to be re-added to the output, resulting in “X̂.” Conversely, the enhanced SE-ResNet module, depicted on the right, starts with the same residual transformation. Following this, the feature map, represented as W x H x C (width, height, and channel dimension), is subject to the SENet’s “Global pooling,” condensing its spatial dimensions. In the architecture of our SENet-enhanced ResNet50 model, particular emphasis is placed on the channel dimension (“C”) in the context of CT images, typically represented in grayscale. This focus on the channel dimension is pivotal, as the SENet mechanism aims to adaptively recalibrate channel-wise feature responses, enhancing the model’s ability to discern subtle variations in grayscale indicative of fractures. Unlike conventional RGB images, where three channels (C = 3) represent color information, CT images usually comprise a single channel (C = 1), underscoring the importance of channel-wise attention in amplifying relevant features for fracture detection. It is imperative to clarify that the SENet’s design inherently targets the channel dimension without altering the spatial dimensions (width “W” and height “H”) of the feature maps. This specificity ensures that the recalibration process enriches the depth of information captured within the channels, fostering a model that is finely attuned to the discriminative features essential for identifying ankle fractures in CT scans. Two subsequent Fully Connected (FC) layers then orchestrate the ‘squeeze’ and ‘excite’ functionalities intrinsic to SENet, refining the model’s focus on specific channels. The culmination is the “Scale” operation, recalibrating the post-residual feature map. By strategically embedding these SE modules within the ResNet50 architecture, our hybrid model aimed to harness the best of both worlds: the depth and gradient benefits of ResNet and the channel-wise recalibration prowess of SENet. This fusion was designed to address the unique challenges of our study more proficiently.

### Comparative analysis of adapted ResNet50, ResNet50, and EfficientNetB5

While the incorporation of the Squeeze-and-Excitation Network (SENet) with ResNet50 was the centerpiece of this study, it was essential to benchmark the performance of this enriched model against other well-established architectures. Consequently, two other models were chosen for comparison: the original ResNet50 and the EfficientNetB5.

The EfficientNetB5 model, a member of the EfficientNet family, is noted for its compound scaling method which uniformly scales all dimensions of depth, width, and resolution using a fixed set of scaling coefficients. This approach was inspired by the observation that the relationship between different dimensions in neural networks is not arbitrary. Rather, it requires a carefully balanced trade-off. Boasting a higher number of layers and parameters compared to its predecessors in the EfficientNet series, EfficientNetB5 provides an impressive balance between computational efficiency and model performance [26].

For the purposes of this study, the same dataset was employed to train all three models: the adapted ResNet50, the original ResNet50 and the EfficientNetB5. The training regimen was held consistent across all models. An initial learning rate of 0.001 was employed, which was reduced by a factor of 10 whenever the validation loss plateaued for more than five epochs. This strategy ensured that each model could hone in on the optimal weights as training progressed. All models were trained for a total of 20 epochs, utilizing the Adam optimizer [27]. The chosen batch size was 32, and the models were trained using a categorical cross-entropy loss function, given the classification nature of the task. Following the training phase, the performance metrics of accuracy, precision, recall, and F1-score were calculated for each model on a separate test dataset.

### Integration and implementation of Grad-CAM technology

Grad-CAM, which stands for Gradient-weighted Class Activation Mapping, has garnered significant attention in recent years as an interpretability tool for convolutional neural networks (CNNs). At its core, Grad-CAM provides visual explanations of the areas within an input image that are vital for predictions made by CNN models [28]. This technique operates by leveraging the gradients of any target concept flowing into the model’s final convolutional layer to produce a coarse localization map, which highlights the important regions in the image for predicting the concept. In this research, the integration of Grad-CAM with our adapted ResNet50 model was executed to enhance the diagnostic capabilities of the model. This decision was underpinned by the belief that while a high classification accuracy is indispensable, understanding why and how a model makes its decisions can significantly augment its value, especially in the field of medical imaging where such insights can aid clinical professionals in corroborating their findings.

The implementation process began by identifying the final convolutional layer in our adapted ResNet50 model. This layer was chosen because it contains high-level feature maps that capture the most discriminative features of the input images. Once identified, the gradients of the predicted class with respect to the feature maps of this convolutional layer were computed. These gradients were then globally average-pooled to obtain the neuron importance weights. By applying a weighted combination of these neuron importance weights with the feature maps and subsequently passing this through a ReLU activation function, the Grad-CAM heatmaps were produced [29]. These heatmaps were then overlaid on the original input images to visually highlight the regions that contributed most significantly to the model’s decision. The coarse Grad-CAM maps were upscaled using bilinear interpolation to match the resolution of the input images. This ensured that the highlighted regions were precisely aligned with the relevant structures in the ankle.

## Results

The systematic evaluation of the performance of different deep learning models in the context of ankle fracture identification offered enlightening insights. The graphical representations in the provided figures provide a comprehensive understanding of the comparative performance of the three models: ResNet50, EfficientNetB5, and the Adapted ResNet50 with SENet capabilities.

### Training convergence and model accuracy over epochs

**Fig. 4:**
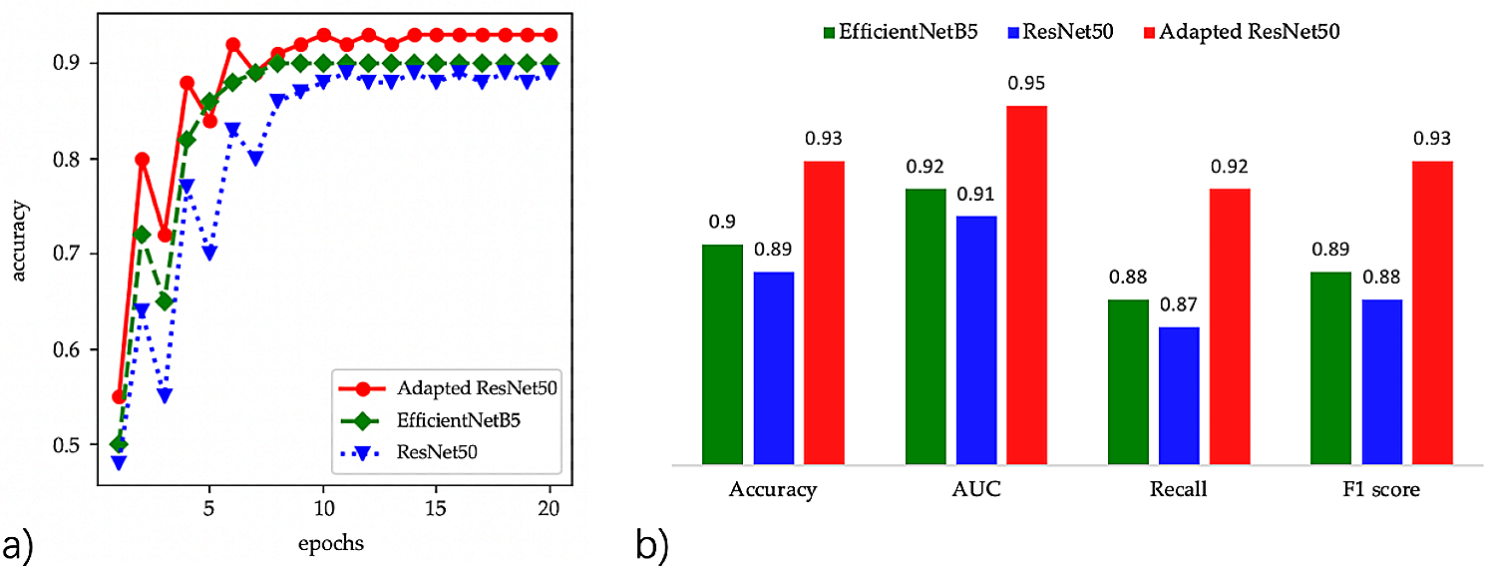
Comparative performance analysis of deep l Models for ankle fracture identification; 4**a**) Training accuracy evolution over epochs; 4**b**) Comparative evaluation of model metrics: accuracy, AUC, Recall, and F1 score

The training accuracy over the epoch progression for each of the models was elucidated in Fig. 4a. Initially, all three models exhibited an evident increase in their training accuracy during the first few epochs. This early surge underscores the rapid learning capabilities of these deep architectures, especially when presented with a well-curated dataset.

The Adapted ResNet50 model (represented by red circles) reached an accuracy just above 0.7 within the first epoch. Its performance continued to rise steeply until around the fifth epoch, post which its progress began to decelerate. By the 20th epoch, its accuracy stabilize by 0.93, indicating its robustness and adaptability in fracture identification. The EfficientNetB5 model’s performance (depicted by green diamonds) evolved similarly, albeit starting from a lower initial accuracy, slightly above 0.6. This model’s accuracy growth rate slowed around the tenth epoch and stabilize at 0.9 by the 20th epoch. ResNet50’s trajectory (illustrated by blue triangles) commenced just below 0.7. Interestingly, its accuracy trajectory mirrored that of the Adapted ResNet50 closely, but it always lagged marginally. This trend persisted, with ResNet50 achieving a stabilization at 0.89, but lower than its adapted counterpart.

### Comparative performance metrics

Figure 4b presented a detailed comparison of the performance metrics for each model. When assessing accuracy, the Adapted ResNet50 model led the cohort with a score of 0.93, substantiating its enhanced capabilities. The EfficientNetB5 followed closely with 0.90, while the original ResNet50 model recorded an accuracy of 0.89. The Area Under the Curve (AUC) metric, often indicative of a model’s capability to distinguish between classes, was also analyzed. The Adapted ResNet50 once again emerged at the forefront with an AUC of 0.95. EfficientNetB5 and ResNet50 closely trailed with scores of 0.92 and 0.91, respectively. Recall, which evaluates the true positive rate of the models, offered an interesting perspective. The Adapted ResNet50 achieved the highest recall of 0.92, while EfficientNetB5 scored 0.88 and ResNet50 scored 0.87. Lastly, the F1 score, a harmonic mean of precision and recall, was assessed. The Adapted ResNet50 secured the top position with a score of 0.93, underscoring its balanced performance. The EfficientNetB5 recorded an F1 score of 0.89, and the ResNet50 rounded off the evaluation with an F1 score of 0.88.

The results unequivocally emphasized the superior performance of the Adapted ResNet50 model across all the evaluated metrics. Its integration with SENet capabilities appeared to have endowed it with enhanced discernment, particularly evident in its training convergence rate and final accuracy.

### Grad-CAM visualization analysis of the adapted ResNet50 model on ankle fracture CT images

The integration of the Grad-CAM visualization technique provided a comprehensive understanding of the areas where the adapted ResNet50 model was particularly attentive when discerning ankle fractures in CT images. This visualization not only aided in offering clarity on the model’s decision-making process but also served as a platform to contrast its performance against the expertise of orthopedic specialists.

**Fig. 5:**
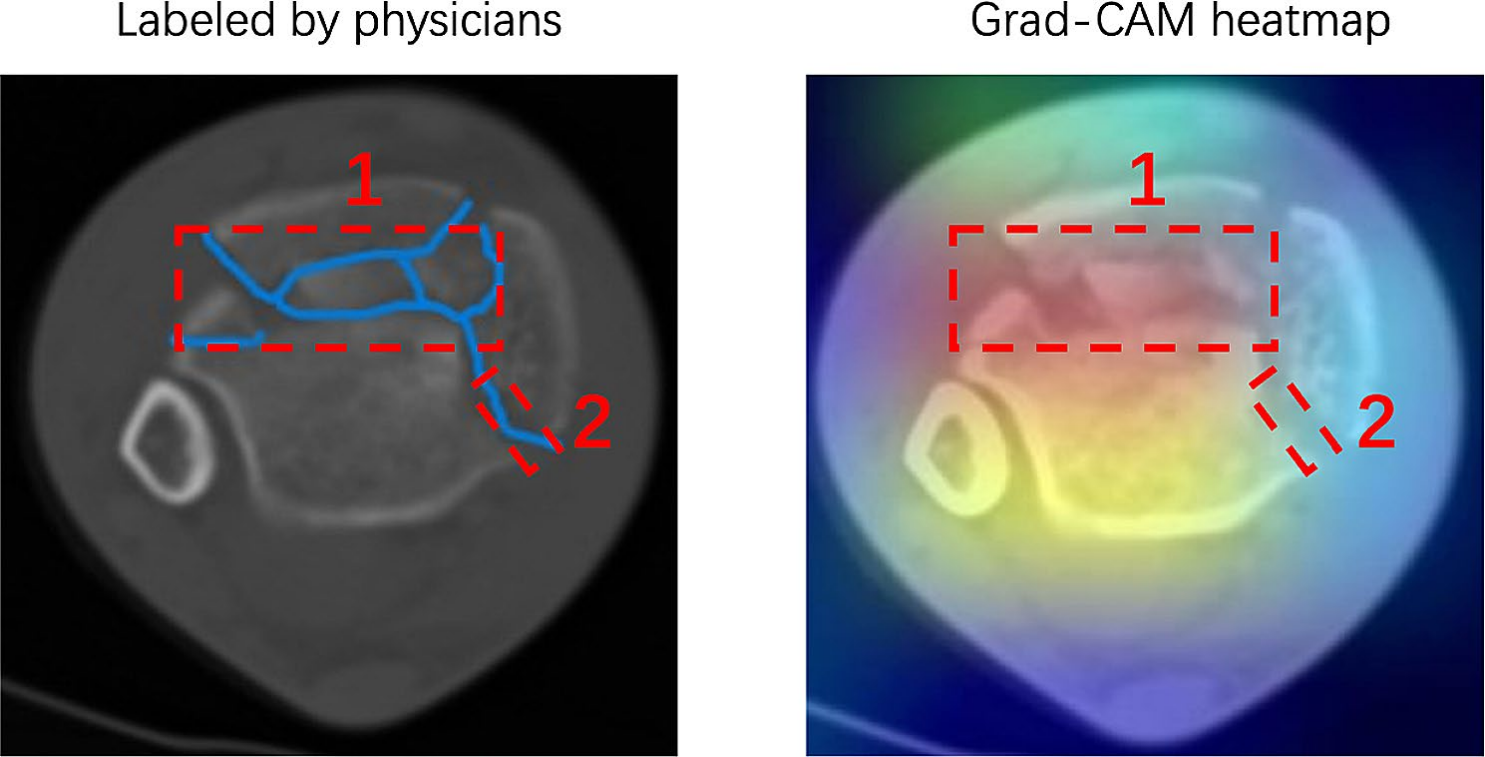
Comparison of orthopedic experts’ annotations and Grad-CAM heatmap on a CT image of an ankle fracture

In the experiment, a representative CT image showcasing an ankle fracture was chosen for analysis. Three orthopedic experts with significant experience in the field were then solicited to demarcate the regions they believed to be indicative of fractures. Their collective annotations provided a benchmark against which the predictions of the adapted ResNet50 model could be weighed. As can be observed in the presented Fig. 5, two principal areas were marked by the orthopedic experts, labeled as area 1 and area 2. Area 1, positioned towards the upper segment of the image, showcased a pronounced overlap between the fracture features recognized by the experts and the regions illuminated on the Grad-CAM heatmap. This congruence underscores the model’s competence in identifying and aligning with the insights of the specialists, suggesting a robust performance in this region. However, a divergent pattern was noted in area 2, located on the lower right quadrant of the image. Here, the experts had identified certain fracture characteristics which the Grad-CAM heatmap failed to highlight. This discrepancy implies that the adapted ResNet50 model might not be capturing all the nuanced features of the fracture in this specific region. This observation suggests an underfitting tendency of the model in certain scenarios, indicating the necessity for further optimization or training to better capture the intricacies of ankle fractures.

## Discussion

The systematic evaluation and comparison of deep learning models in the arena of medical imaging, specifically for the identification of ankle fractures using CT images, yielded notable insights. The core innovation of this research was the development and integration of the Adapted ResNet50 model with SENet capabilities. In the burgeoning field of deep learning, ResNet architectures have consistently been acknowledged for their prowess in mitigating vanishing gradient issues and enabling the training of deeper networks [25]. However, it was discerned that while traditional ResNet architectures brought commendable capabilities to the table, there remained scope for enhancement.

This realization steered our efforts toward adapting the standard ResNet50 model by incorporating SENet capabilities. The rationale behind this integration was rooted in the SENet’s distinctive ability to refine channel-wise feature responses adaptively [23]. By doing so, it could recalibrate the features more effectively, which was surmised to amplify the discriminatory capabilities of the model, especially in tasks as nuanced as ankle fracture identification.

In the presented results, the benefits of SENet integration into the ResNet50 architecture were clearly demonstrated, with the Adapted ResNet50 model showing significant improvements over its peers in our study, notably the original ResNet50 and EfficientNet models, across multiple metrics. While these findings highlight the potential of SENet capabilities in enhancing model performance, we acknowledge that our comparison was focused on a limited set of architectures. The observed enhancements, while substantial within this context, invite further exploration against a broader spectrum of models to fully validate the generalizability of our results.

It’s noteworthy that other architectures, specifically EfficientNetB5, were also under scrutiny. EfficientNets have been revered for their compound scaling method, balancing depth, width, and resolution [26]. However, even with its inherent sophistication, it was observed that the model was marginally eclipsed by our Adapted ResNet50, especially in terms of convergence rate and final accuracy.

In comparison with previous literature on the use of deep learning for ankle fracture detection, such as the study in 2021 [30] that achieved high sensitivity and specificity using radiographs, our research contributes to the expanding body of knowledge by exploring the use of CT images and SENet-enhanced ResNet50. The referenced study demonstrated the effectiveness of employing Inception V3 and ResNet50 with radiographs, highlighting the potential of deep learning methods in accurately assessing fractures with high precision. Notably, their use of the Danis-Weber classification method and the comparison of single-view versus three-view radiographs for training DCNNs provided valuable insights into optimizing image selection for deep learning algorithms. Our study’s focus on CT images, known for their detailed visualization of bone structures, alongside the novel integration of SENet, aims to further enhance the diagnostic accuracy and interpretability of deep learning models. This approach underscores the diversity of methodologies and imaging modalities that can be leveraged to improve fracture detection. The high performance of DCNNs in the previous study, with sensitivity and specificity reaching up to 98.7% and 98.6% respectively using Inception V3, sets a benchmark for our work and others in the field [30]. Our findings contribute to this ongoing dialogue by suggesting that enhancements such as SENet can provide significant improvements, particularly in the context of CT-based fracture detection, which presents different challenges and opportunities compared to radiograph-based assessments. Future research could explore the comparative effectiveness of different deep learning architectures across various imaging modalities, potentially incorporating multimodal approaches to achieve the highest diagnostic accuracy.

Incorporating the Grad-CAM visualization technique was another pivotal aspect of this study. This technique illuminated the regions of focus for our adapted model, providing a visual corroboration of its decisions. While this in itself is not novel, using it to juxtapose the model’s inferences with expert annotations was an enlightening endeavor. This comparative analysis not only strengthened the credibility of the model’s predictions but also highlighted areas where human expertise and machine predictions diverged. In addressing the inherent limitations of Grad-CAM for practical applications in medical imaging, particularly its occasional misalignment with clinically relevant areas, we emphasize the importance of a multi-faceted approach. This includes rigorous model training and expert validation of visualization outputs. These strategies collectively enhance the practical utility of Grad-CAM in our study, ensuring that the visualizations it provides are both accurate and clinically meaningful. Our commitment to these practices reflects our goal to bridge the gap between deep learning technology and its application in enhancing diagnostic accuracy and efficiency in orthopedics.

The implications of our findings are manifold. For healthcare professionals, especially orthopedic specialists, tools such as the Adapted ResNet50 model can be invaluable adjuncts. They can bolster diagnostic accuracy, expedite the decision-making process, and potentially enhance patient outcomes. From the perspective of AI research, this study exemplifies how even established architectures like ResNet can be reimagined and enhanced, pushing the boundaries of what’s achievable.

In the realm of the identification of ankle fractures, the incorporation of the Squeeze-and-Excitation Network (SENet) mechanism represents a significant architectural advancement. The SENet mechanism, known for its channel-wise attention functionality, allows for the adaptive recalibration of channel-wise feature responses. This recalibration enables the model to prioritize more relevant features over less informative ones, thereby improving the accuracy and reliability of fracture detection.

To elucidate the impact of the SENet mechanism on the learned features of our enhanced ResNet50 model, we embarked on a comprehensive interpretation strategy. Initially, through feature visualization, we observed how the inclusion of SENet blocks altered the network’s focus. Activation maps generated before and after applying SENet blocks unveiled a discernible shift in attention towards regions critical for accurate fracture identification. This shift not only underscores the SENet’s capability to refine the model’s interpretability but also highlights its utility in emphasizing salient features for the task at hand.

Further quantitative analysis was conducted via ablation studies, where the performance metrics of models with and without the SENet blocks were compared. These studies revealed that the incorporation of SENet significantly enhances the model’s discriminative power, as evidenced by improvements in accuracy, precision, and recall metrics. Such findings quantitatively substantiate the SENet mechanism’s vital role in bolstering the model’s performance.

Through these interpretative efforts, it became evident that the SENet mechanism significantly refines the model’s focus, leading to more accurate and dependable fracture identification. This adaptive recalibration of channel-wise feature responses not only enhances the model’s accuracy but also its interpretability, offering a profound understanding of the complex patterns characteristic of ankle fractures.

Despite the comprehensive nature of the study and the promising results observed, several limitations warrant acknowledgment. Firstly, the dataset predominantly relied upon a single source, potentially introducing biases and limiting the generalizability of findings to broader contexts. Additionally, the exclusive focus on CT images, without integrating complementary modalities like X-rays or MRIs, might also restrict the holistic understanding of ankle fractures. Foremore, in this study, our focus was on the detection of ankle fractures using SENet-enhanced ResNet50, without classifying them according to the Danis-Weber or other orthopedic systems. The Danis-Weber classification is crucial for clinical decision-making but was not part of our initial model training criteria. Our aim was to first establish a reliable method for fracture detection. Future work will explore extending our model to classify fractures based on established systems like Danis-Weber, enhancing both diagnostic accuracy and clinical utility.

## Conclusion

The study provides a meticulous analysis of the capabilities of various deep learning models, specifically focusing on their performance in the detection of ankle fractures. Among the evaluated models, the Adapted ResNet50 with SENet capabilities unequivocally emerged as the top performer across multiple metrics, including accuracy, AUC, recall, and the F1 score. Its rapid training convergence and a final accuracy of 0.93 underscore the potential advantages of integrating SENet capabilities into the ResNet50 architecture. The Grad-CAM visualization technique employed with the Adapted ResNet50 model further illuminated the areas of focus when discerning fractures in CT images. A comparison with expert annotations showcased the model’s competence in identifying fractures, with pronounced overlap in certain areas. In light of these findings, the Adapted ResNet50 model represents a promising tool in the realm of orthopedic diagnostics.

## Data Availability

The datasets used and/or analysed during the current study are available from the corresponding author on reasonable request.
